# Comparative Chloroplast Genomes of *Nicotiana* Species (Solanaceae): Insights Into the Genetic Variation, Phylogenetic Relationship, and Polyploid Speciation

**DOI:** 10.3389/fpls.2022.899252

**Published:** 2022-07-04

**Authors:** Shuaibin Wang, Junping Gao, Haoyu Chao, Zhaowu Li, Wenxuan Pu, Yaofu Wang, Ming Chen

**Affiliations:** ^1^Department of Bioinformatics, State Key Laboratory of Plant Physiology and Biochemistry, College of Life Sciences, Zhejiang University, Hangzhou, China; ^2^Tobacco Research Institute of Technology Centre, China Tobacco Hunan Industrial Corporation, Changsha, China; ^3^State Key Laboratory of Crop Genetics and Germplasm Enhancement, Nanjing Agricultural University, Nanjing, China

**Keywords:** *Nicotiana* L., chloroplast genome, genetic variation, phylogenetic relationship, divergence time estimation, polyploid speciation

## Abstract

*Nicotiana* L. is a genus rich in polyploidy, which represents an ideal natural system for investigating speciation, biodiversity, and phytogeography. Despite a wealth of phylogenetic work on this genus, a robust evolutionary framework with a dated molecular phylogeny for the genus is still lacking. In this study, the 19 complete chloroplast genomes of *Nicotiana* species were assembled, and five published chloroplast genomes of *Nicotiana* were retrieved for comparative analyses. The results showed that the 24 chloroplast genomes of *Nicotiana*, ranging from 155,327 bp (*N. paniculata*) to 156,142 bp (*N. heterantha*) in size, exhibited typical quadripartite structure. The chloroplast genomes were rather conserved in genome structure, GC content, RNA editing sites, and gene content and order. The higher GC content observed in the IR regions could be a result of the presence of abundant rRNA and tRNA genes, which contained a relatively higher GC content. A total of seven hypervariable regions, as new molecular markers for phylogenetic analysis, were uncovered. Based on 78 protein-coding genes, we constructed a well-supported phylogenetic tree, which was largely in agreement with previous studies, except for a slight conflict in several sections. Chloroplast phylogenetic results indicated that the progenitors of diploid *N. sylvestris, N. knightiana*, and the common ancestor of *N. sylvestris* and *N. glauca* might have donated the maternal genomes of allopolyploid *N. tabacum, N. rustica*, and section *Repandae*, respectively. Meanwhile, the diploid section *Noctiflorae* lineages (*N. glauca*) acted as the most likely maternal progenitor of section *Suaveolentes*. Molecular dating results show that the polyploid events range considerably in ~0.12 million (section *Nicotiana*) to ~5.77 million (section *Repandae*) years ago. The younger polyploids (*N. tabacum* and *N. rustica*) were estimated to have arisen ~0.120 and ~0.186 Mya, respectively. The older polyploids (section *Repandae* and *Suaveolentes*) were considered to have originated from a single polyploid event at ~5.77 and ~4.49 Mya, respectively. In summary, the comparative analysis of chloroplast genomes of *Nicotiana* species has not only revealed a series of new insights into the genetic variation and phylogenetic relationships in *Nicotiana* but also provided rich genetic resources for speciation and biodiversity research in the future.

## Introduction

The *Nicotiana* L., after *Solanum, Cestrum, Physalis*, and *Lycium*, is the fifth-largest genus of Solanaceae, a megadiverse family that includes many economically important crop plants such as tomato, potato, and eggplant (Clarkson et al., 2004). The genus *Nicotiana* has 75 naturally occurring species (40 diploids and 35 allopolyploids), which were subdivided into three subgenera and 14 sections (Rustica: *Paniculatae, Thyrsiflorae, Rusticae*; Tabacum: *Tomentosae, Genuinae*; Petunioides: *Undulatae, Trigonophyllae, Sylvestres, Repandae, Notctiflorae, Acuminatae, Bigelovianae, Nudicaules, Suaveolentes*) by Goodspeed and Knapp (Goodspeed, 1956; Knapp et al., 2004). The *Nicotiana* species occurred largely in the Americas and Australia, with one (*N. africana*) in Africa and another (*N. fragrans*) in the South Pacific Ocean (Aoki and Ito, 2000), however, the cultivated tobacco (*N. tabacum* and *N. rustica*) had been spread worldwide by humans (Knapp et al., 2004). The hypothesized ancestral basic chromosome number is *x* = 12, and the polyploidy and aneuploidy have occurred independently several times during the evolution of the *Nicotiana* species (Aoki and Ito, 2000). Approximately half of the *Nicotiana* species were thought to be natural tetraploid species (Goodspeed, 1956). In comparison to other plants, *Nicotiana* species are, therefore, ideally positioned to take advantage of recent advances in speciation, biodiversity, and phytogeography (Aoki and Ito, 2000). Phylogenetic analysis among *Nicotiana* species, for evolutionary biological research, has been performed based on the internal transcribed spacer regions (ITS) (Komarnyts'kyi et al., 1998), chloroplast-expressed glutamine synthetase gene (ncpGS) (Clarkson et al., 2010), maturase K (matK) gene (Aoki and Ito, 2000; Bally et al., 2021), multiple chloroplast DNA regions (Clarkson et al., 2004), and random amplified polymorphic DNA (RAPD) of genomic DNA (Khan and Narayan, 2007). Results of molecular phylogenetic comparison have provided a greater understanding of the evolutionary processes underlying genome differentiation in *Nicotiana* and helped solve several unresolved problems in the evolution of this genus. Phylogenetic studies also have shown that these allopolyploid species in the genus *Nicotiana* were formed at ~0.2 million (*N. rustica* and *N. tabacum*) to more than 10 million years ago (section *Suaveolentes*) (Clarkson et al., 2004; Leitch et al., 2008). However, the previous results of phylogenetic relationships lacking support could be attributed to insufficient informative sites. Further study is needed to examine the origin and speciation of the genus *Nicotiana*.

The development of high-throughput sequencing technologies and constantly optimized assembly strategies has facilitated rapid progress in the field of chloroplast genetics and genomics (Daniell et al., 2016). Since the first chloroplast genome was sequenced for *N. tabacum* in 1986 (Shinozaki et al., 1986), over one thousand complete chloroplast genome sequences from land plants have been made available in the National Center for Biotechnology Information (NCBI) organelle genome database (https://www.ncbi.nlm.nih.gov/genome/browse#!/organelles/). In angiosperms, the chloroplast genomes are typically circular (though linear forms have also been observed) (Oldenburg and Bendich, 2016) and the size of chloroplast genomes and their gene arrangement are generally highly conserved, with a range from 120 to 150 kb in length (Palmer, 1991). Angiosperm chloroplast genomes commonly contain ~130 genes encoding up to 80 unique proteins, four ribosomal RNA (rRNA) genes, and ~30 transfer RNA (tRNA) (Daniell et al., 2016). Throughout evolution, increasing numbers of chloroplast genes have been transferred to the genome in the cell nucleus (Kleine et al., 2009). As a result, proteins encoded by nuclear DNA have become essential to chloroplast function (Bryant et al., 2010). Compared with nuclear genomes, chloroplast genomes of land plants have highly conserved circular DNA molecules with two inverted repeat (IR) regions (IRa and IRb) (identical but in opposite orientations) that are separated by small and large single-copy regions (SSC and LSC) (Cui et al., 2006). The whole chloroplast genomes have been widely utilized for reconstructing phylogenetic relationships, DNA barcoding, and the development of molecular markers (Moore et al., 2010; Barrett et al., 2016; Gao et al., 2018; Liu et al., 2021). Using complete chloroplast genomes, many previous studies have performed genetic variations and related phylogenetic analyses in the genus *Citrus, Crataegus, Chlorophytum*, and their relatives (Carbonell-Caballero et al., 2015; Munyao et al., 2020; Wu et al., 2021). Despite the utility of chloroplast genomes for determining hybridization events and phylogenetic relationships between species (Brock et al., 2022), only several complete chloroplast genomes have previously existed for *Nicotiana*. Until now, the complete chloroplast genomes have been reported for 11 species in the genus *Nicotiana*, including *N. attenuata, N. glauca, N. knightiana, N. obtusifolia, N. otophora, N. paniculata, N. rustica, N. sylvestris, N. tabacum, N. tomentosiformis*, and *N. undulata* (Shinozaki et al., 1986; Yukawa et al., 2006; Asaf et al., 2016; Mehmood et al., 2020). Based on the sequences of chloroplast genomes, an ancestor of *N. sylvestris* (2*n* = 2*x* = 24, section *Sylvestres*) was identified uncontroversially as the maternal donor (S-genome) of cultivated tobacco (*N. tabacum*, 2*n* = 4*x* = 48, section *Nicotiana*) (Yukawa et al., 2006). The previous phylogenetic relationships in *Nicotiana* species were based on multiple chloroplast DNA regions (Clarkson et al., 2004), which did not appear to fully resolve the phylogenetic relationship of the *Nicotiana* species. More recently, the comparative analysis of chloroplast genomes among five *Nicotiana* species was performed (Mehmood et al., 2020), but taxon sampling was too sparse to make major conclusions about the evolution of many groups.

In this study, we performed a comparative analysis of the 24 complete chloroplast genomes of the genus *Nicotiana* including five earlier published chloroplast genomes (Yukawa et al., 2006; Gao et al., 2016; Poczai et al., 2017) and the 19 chloroplast genomes of *Nicotiana* species, subspecies, and varieties (*N. tabacum* cv. Basma Xanthi, *N. tabacum* cv. K326, *N. glauca, N. benthamiana, N. heterantha, N. cavicola, N. simulans, N. rosulata* subsp. rosulata, *N. occidentalis* subsp. obliqua, *N. occidentalis* subsp. occidentalis, *N. nesophila, N. stocktonii, N. repanda, N. nudicaulis, N. rustica, N. knightiana*, and *N. paniculata*) newly assembled in this study. Our main objects were to (1) understand deeply interspecific variation within the chloroplast genomes, (2) identify variation hotspot regions as candidate sequences for species identification and further speciation studies in *Nicotiana* species, (3) resolve well-supported phylogenetic relationships and recognize the origin and evolution of the allotetraploid species among the genus *Nicotiana*, (4) estimate the divergence times of the *Nicotiana* species. These results not only provided a series of new insights into genetic variation and phylogenetic relationships but also enabled us to identify promising germplasm resources for the genetic improvement of genus *Nicotiana*.

## Materials and Methods

### Data Sources, Assembly, and Annotation of Chloroplast Genomes

Totally 19 accessible DNA sequencing data of *Nicotiana* species were received from the Sequence Read Archive database of NCBI (https://www.ncbi.nlm.nih.gov/) including *N. tabacum* cv. Basma Xanthi (SRR955782), *N. tabacum* cv. K326 (SRR955771), *N. glauca* (SRR6320052), *N. benthamiana* (SRR7540368), *N. heterantha* (SRR8666768), *N. cavicola* (SRR7692018), *N. simulans* (SRR8666472), *N. rosulata* subsp. rosulata (SRR8666798), *N. occidentalis* subsp. obliqua (SRR8666800), *N. occidentalis* subsp. *occidentalis* (SRR8666801), *N. nesophila* (SRR4046065), *N. stocktonii* (SRR4046066), *N. repanda* (SRR453021), *N. nudicaulis* (SRR452996), *N. rustica* (SRR8173847), *N. knightiana* (SRR8169728), *N. paniculata* (SRR8173261), *N. obtusifolia* (SRR3592436), and *N. otophora* (SRR954962) (Table 1).

**Table 1:** Sample information and summary of 24 chloroplast genome characteristics of *Nicotiana* species.

**Species**	**Data sources**	**Total length (bp)**		**LSC length (bp)**		**SSC length (bp)**		**IRs length (bp)**		**Gene regions (bp)**	**Intergenic regions (bp)**
		**Length (bp)**	**GC content**	**Length (bp)**	**GC content**	**Length (bp)**	**GC content**	**Length (bp)**	**GC content**		
*N. tabacum* cv. Basma Xanthi	SRR955782	155,942	37.85%	86,686	35.95%	18,572	32.06%	25,342	43.22%	109,374	46,568
*N. tabacum* cv. K326	SRR955771	156,026	37.84%	86,770	35.93%	18,572	32.06%	25,342	43.22%	109,457	46,569
*N. tabacum* cv. TN90	KU199713	155,992	37.84%	86,814	35.93%	18,572	32.06%	25,303	43.26%	109,405	46,587
*N. sylvestris*	NC_007500	155,941	37.85%	86,685	35.95%	18,572	32.06%	25,342	43.22%	109,375	46,566
*N. glauca*	SRR6320052	156,054	37.83%	86,657	35.96%	18,587	32.03%	25,405	43.15%	109,355	46,699
*N. benthamiana*	SRR7540368	155,726	37.86%	86,319	35.99%	18,569	32.04%	25,419	43.15%	109,347	46,379
*N. heterantha*	SRR8666768	156,142	37.75%	86,521	35.89%	18,573	31.94%	25,524	43.01%	109,381	46,761
*N. cavicola*	SRR7692018	155,851	37.86%	86,341	35.99%	18,420	32.15%	25,545	43.08%	109,516	46,335
*N. simulans*	SRR8666472	155,803	37.84%	86,375	35.96%	18,582	32.02%	25,423	43.14%	109,387	46,416
*N. rosulata* subsp. *rosulata*	SRR8666798	155,966	37.79%	86,348	35.94%	18,582	32.03%	25,518	43.01%	109,338	46,628
*N. occidentalis* subsp. *obliqua*	SRR8666800	155,880	37.81%	86,417	35.94%	18,579	32.03%	25,442	43.12%	109,379	46,501
*N. occidentalis* subsp. *occidentalis*	SRR8666801	155,874	37.82%	86,459	35.92%	18,583	32.02%	25,416	43.16%	109,386	46,488
*N. nesophila*	SRR4046065	155,577	37.91%	86,443	36.02%	18,576	32.18%	25,279	43.23%	109,146	46,431
*N. stocktonii*	SRR4046066	155,480	37.92%	86,340	36.05%	18,582	32.17%	25,279	43.23%	109,144	46,336
*N. repanda*	SRR453021	155,454	37.90%	86,236	36.03%	18,538	32.18%	25,340	43.19%	109,159	46,295
*N. nudicaulis*	SRR452996	155,538	37.90%	86,486	36.01%	18,566	32.13%	25,243	43.26%	109,176	46,362
*N. rustica*	SRR8173847	155,336	37.87%	85,974	35.99%	18,552	32.12%	25,405	43.16%	109,320	46,016
*N. knightiana*	SRR8169728	155,337	37.87%	85,977	35.98%	18,552	32.11%	25,404	43.17%	109,324	46,013
*N. paniculata*	SRR8173261	155,327	37.88%	85,972	35.99%	18,549	32.14%	25,403	43.17%	109,314	46,013
*N. undulata*	JN563929	155,863	37.88%	86,634	35.99%	18,569	32.12%	25,330	43.23%	109,355	46,508
*N. attenuata*	MG182422	155,914	37.86%	86,514	35.99%	18,526	32.06%	25,437	43.17%	109,427	46,487
*N. obtusifolia*	SRR3592436	155,811	37.79%	86,597	35.87%	18,566	31.90%	25,324	43.23%	109,347	46,464
*N. otophora*	SRR954962	155,912	37.76%	86,609	35.83%	18,499	31.96%	25,402	43.15%	109,288	46,624
*N. tomentosiformis*	AB240139	155,745	37.79%	86,393	35.88%	18,496	31.96%	25,428	43.16%	109,404	46,341

The Fastq files of Illumina sequence data were extracted from SRA files using the SRA Toolkit (https://trace.ncbi.nlm.nih.gov/Traces/sra/sra.cgi?view=software). Low-quality reads with a phred score < 20 and length < 50 were removed using fastp (Chen et al., 2018). The remaining high-quality reads were used to assemble chloroplast genomes using NOVOplasty v4.3.1 (Dierckxsens et al., 2017) with *N. sylvestris* (GenBank No. NC_007500) as a reference. In addition, for failed assembled samples from NOVOplasty, we also used SPAdes v3.15 (Bankevich et al., 2012) with *K*-mer lengths of 87, 93, and 97 to assemble high-quality fragments, and the assembled contigs were further checked using BLAST search (Camacho et al., 2009) against the *N. sylvestris* chloroplast genome. The related position and direction of each contig were manually adjusted according to the reference genome (*N. sylvestris*). Finally, the chloroplast genomes were further polished to correct errors and ambiguous regions using Pilon (Walker et al., 2014).

The complete chloroplast genomes were annotated using GeSeq (Tillich et al., 2017). Following annotation, the start/stop codons and the position of introns were manually inspected and curated according to the reference chloroplast genome in the SnapGene software (https://snapgene.com). The annotation of the transfer RNA (tRNA) genes was verified by tRNAscan-SE version 2.0 (Lowe and Eddy, 1997) with default settings. The boundary of the large single-copy (LSC) region, small single-copy (SSC) region, and a pair of inverted repeats (IRs) regions for each chloroplast genome was verified using BLAST (Camacho et al., 2009). To verify the assembly results, the depth of coverage was determined by mapping all reads to each finished chloroplast genome with BWA (Li, 2013) and visualized with Circos (Krzywinski et al., 2009).

### RNA Editing Prediction and Genetic Variation Analyses

The online software PREP-cp (Putative RNA Editing Predictor of Chloroplast) (Mower, 2009) was used with default settings to determine putative RNA editing sites. Clean read sets were then separately mapped to the *N. sylvestris* reference genome (one IR region removed) using BWA (Li, 2013). Variant calling was performed using FreeBayes v.1.3.6 (Garrison and Marth, 2012) with the parameter –ploidy 1 and VCF files were filtered using vcffilter of vcflib v1.0.3 (https://github.com/vcflib/vcflib) with the parameter -f “QUAL > 20”. SNPs and InDels were, respectively, extracted by vcffilter -f “TYPE = snp” and “(TYPE = ins | TYPE = del)”. SNPs and InDels statistics were calculated using vcfstats of vcflib. Microsatellite repeats (SSRs) within the chloroplast genomes of *Nicotiana* species were detected using MISA software (Beier et al., 2017) by setting the minimal repeat number of 7 for mononucleotide repeats, 4 for di-, and 3 for tri-, tetra-, penta- and hexanucleotide SSRs. We also used vmatch software (Kurtz et al., 2001) with the following parameters: minimal repeats length was set to 30 bp, hamming distance to 3 for scanning and visualizing forward (F) and palindromic (P) repeats in the chloroplast genome of *Nicotiana* species. Tandem repeats were found with the trf (tandem repeats finder) using default parameters (Benson, 1999). The visualization of the circular maps of the chloroplast genomes, the GC content, and the densities of nucleotide variability (Pi), SNPs and InDels (i.e., the number of SNPs or InDels counted for every consecutive 500 bp blocks) over the entire chloroplast genomes were performed using Circos (Krzywinski et al., 2009).

### Phylogenetic Analyses

In this study, total of 24 chloroplast genomes of *Nacotiana* species was used to infer the phylogenetic relationships. The chloroplast genome of *Solanum agnewiorum* (GenBank No. NC_039416) (Aubriot et al., 2018) was set as outgroup. All 78 protein-coding genes were extracted using a customized Python script from each chloroplast genome. For phylogenetic analysis, the coding alignments were constructed using MAFFT v7.490 (Katoh and Standley, 2013) with default parameters and concatenated by AMAS (Borowiec, 2016) with a concatenated matrix length of 68,484 bp. The best-fitting model (TVM+I+G4) was determined by model test-ng (Darriba et al., 2019) according to the Akaike information criterion (AIC). Maximum likelihood (ML) analyses were performed with raxml-ng (Kozlov et al., 2019) using the ultrafast bootstrap approximation with 1,000 replicates. The phylogeny tree was visualized using FigTree v1.4.4 software (http://tree.bio.ed.ac.uk/software/figtree/).

### Divergence Time Estimates

The relative divergence times of the *Nicotiana* species were estimated using BEAST v2.6.6 (Suchard et al., 2018) optimized for OpenGL graphics. The concatenated analysis of 78 protein-coding genes from 24 *Nicotiana* chloroplast genomes was run for 20 million generations with sampling every 1,000 replication under the BEAST equivalents of the JModelTest2 models (Darriba et al., 2012) with six gamma categories. The tree prior used the Calibrated Yule Model (Suchard et al., 2018) with a relaxed log normal clock and site models unlinked. The median time split between the *S. agnewiorum* and *N. undulata* (mean = 25 Myr; standard deviation = 0.5) was used as a temporal constraint to calibrate the BEAST analyses according to the previous studies (Särkinen et al., 2013; Mehmood et al., 2020). The XML output from BEAUTi was sent to BEAST using default parameters. Tracer v1.7.2 (Rambaut et al., 2018) was used to evaluate, ensure convergence and effective sample size (ESS) values, density plots, and trace plots. Tree files were combined, after the removal of 10% burn-in, and a maximum clade credibility tree was constructed using TreeAnnotator v2.6.6 (Suchard et al., 2018) to display median ages and 95% highest posterior density intervals (upper and lower) for each node. The final tree was drawn using FigTree v.1.4.4 software.

## Results

### Basic Characteristics of the Acquired *Nicotiana* Chloroplast Genomes

**Figure 1:**
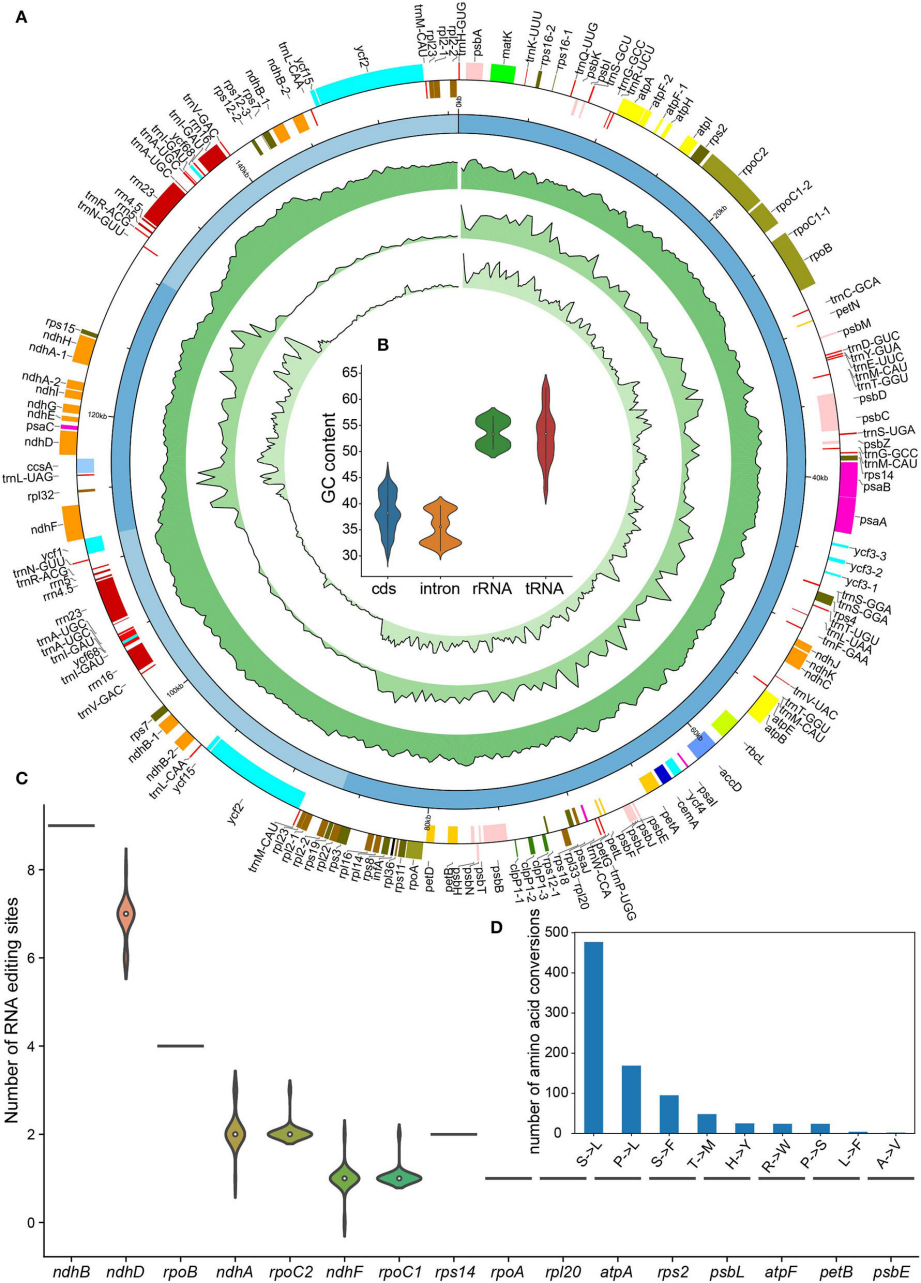
Basic characteristics of the 24 *Nicotiana* chloroplast genomes. **(A)** Circos plot showing basic characteristics of the chloroplast genomes acquired in this study. The gene position, quadripartite structure, GC content, density of variant sites, and nucleotide diversity (Pi) were shown from the outer to inner rings. The outermost rectangles were chloroplast genes belonging to different functional groups that were color-coded. Gene blocks shown on the outside and inside the circle were transcribed clockwise and counterclockwise, respectively. **(B)** The GC content of CDS, intron, tRNA, and rRNA genes among *Nicotiana* chloroplast genomes. **(C)** The number of RNA editing sites among genes of chloroplast genomes. **(D)** The number of amino acid conversions resulting from RNA editing in chloroplast genomes.

The 19 *Nicotiana* chloroplast genomes assembled within this study had this typical quadripartite structure, which was like those earlier published six *Nicotiana* chloroplast genomes (Shinozaki et al., 1986; Asaf et al., 2016; Mehmood et al., 2020) (Figure 1A). Within *Nicotiana* species, the 24 complete chloroplast genomes ranged in size from 155,327 bp for *N. paniculata* to 156,142 bp for *N. heterantha*, and the GC content had a narrow range from 37.75 to 37.92% (Table 1). All chloroplast genomes in the genus *Nicotiana* had a typical circular structure with four junction regions including the LSC region of 85,972–86,814 bp, the SSC region of 18,420–18,587 bp, and the IR regions of 25,342–25,545 bp.

We successfully annotated all chloroplast genomes newly assembled in this study using GeSeq (Tillich et al., 2017). We found that the chloroplast genomes of *Nicotiana* species contained a total of 128 genes, among which there were 37 tRNA genes, 8 rRNA genes, and 83 protein-coding genes (Table 2). As with earlier reports about *Nicotiana* chloroplast genomes (Asaf et al., 2016), a total of 102 unique genes, comprising 78 protein-coding genes, 20 tRNA genes, and four rRNA genes, were detected in each *Nicotiana* chloroplast genome (Table 2). The gene number was the same for all the 24 chloroplast genomes (Figure 1A) and was also in line with all published *Nicotiana* chloroplast genomes so far (Asaf et al., 2016).

**Table 2:** Genes in the 24 chloroplast genomes of *Nicotiana* species.

**Category**	**Group of genes**	**Name of genes**
Self-replication	Large subunit of ribosomal proteins	rpl2^*^ (2), rpl14, rpl16^*^, *rpl20, rpl22, rpl23, rpl32, rpl33, rpl36*
	Small subunit of ribosomal proteins	*rps2, rps3, rps4, rps7* (2), *rps8, rps11, rps12*ª (2), rps14, rps15, rps16^*^, *rps18, rps19*
	DNA dependent RNA polymerase	rpoA, rpoB, rpoC1^*^, *rpoC2*
	rRNA genes	*rrn16* (2), *rrn23* (2), *rrn4.5* (2), *rrn5* (2)
	tRNA genes	trnA^*^ (2), *trnC* (1), *trnD* (1), *trnE* (1), *trnF* (1), trnG^*^ (2), *trnH* (1), trnI^*^ (2), trnK^*^ (1), trnL^*^ (4), *trnM* (4), *trnN* (2), *trnP* (1), *trnQ* (1), *trnR* (3), *trnS* (3), *trnT* (2), trnV^*^ (3), *trnW* (1), *trnY* (1)
Photosynthesis	Photosystem I	*psaA, psaB, psaC, psaI, psaJ*
	Photosystem II	*psbA, psbB, psbC, psbD, psbE, psbF, psbH, psbI, psbJ, psbK, psbL, psbM, psbN, psbT, psbZ*
	NadH oxidoreductase	ndhA^*^, ndhB^*^ (2), *ndhC, ndhD, ndhE, ndhF, ndhG, ndhH, ndhI, ndhJ, ndhK*
	Cytochrome b6/f complex	petA, petB^*^, petD^*^, *petG, petL, petN*
	ATP synthase	atpA, atpB, atpE, atpF^*^, *atpH, atpI*
	Rubisco	*rbcL*
Other genes	Maturase	*matK*
	Protease	*clpP*ª
	Envelop membrane protein	*cemA*
	Subunit AcetylCoA-Carboxylate	*accD*
	c-type cytochrome synthesis gene	*ccsA*
Unknown	Conserved Open reading frames	*ycf1, ycf2* (2), *ycf3*ª, *ycf4*

* *One intron; ªTwo introns; ()gene number*.

Total 13 protein-coding genes (*ccsA, ndhA, ndhD, ndhE, ndhF, ndhG, ndhH, ndhI, psaC, rpl32, rps12, rps15, ycf1*) and one tRNA gene (*trnL*) were distributed in the SSC region, while 62 protein-coding genes and 25 tRNA genes were distributed in the LSC region. Total eight protein-coding genes (*ndhB, rpl2, rpl23, rps12, rps7, ycf15, ycf2, ycf68*), seven tRNA genes (*trnN, trnR, trnA, trnI, trnV, trnL, trnM*), and four rRNA genes (*rrn5, rrn4*.5, *rrn23, rrn16*) distributed in the two IR regions were present in two copies (Figure 1A). Of the 78 protein-coding genes, 12 genes contained one intron (*rpl2, rpl16, rps16, rpoC1, petB, petD, ndhB, ndhA, atpF*) or two introns (*ycf3, clpP, rps12*) in the 24 chloroplast genomes. In addition, six of the identified tRNA genes (*trnK, trnG, trnL, trnV, trnI, trnA*) contained one intron (Table 2). The *rps12* gene in genus *Nicotiana* was recognized as a trans-spliced gene, with the first exon located in the LSC region and the other two exons distributed in the IR regions. The intergenic regions of these genomes ranged within 46,013–46,761 bp in length, accounting for 29.62–29.95% of the total genomes (Table 1).

Similar to earlier studies (Asaf et al., 2016), the overall GC contents of the 24 assembled chloroplast genomes were 37.75–37.92% and the GC contents were not evenly distributed among the different genome regions: IRs (43.01–43.26%) had higher GC content than LSC (35.83–36.05%) and SSC (31.90–32.18%) (Table 1). We also observed the substantial difference in GC content among gene features of *Nicotiana* chloroplast genomes including GC content of CDS, intron, tRNA, and rRNA genes. The tRNA genes (about 53.0%) and rRNA genes (about 53.5%) had higher mean GC content than CDS regions (about 38.3%) and intron regions (about 35.4%) (Figure 1B). Consequently, the IR regions possessed the highest GC content due to an abundance of tRNA and rRNA gene content compared to the rest of the chloroplast genomes (Figure 1A).

For RNA editing analysis, the number of predicted RNA editing sites varied from 35 to 38 (Supplementary Table 1). All these sites were C-to-U conversions and around 91% of these sites were observed on the second base of codon among 16 chloroplast genes of *Nicotiana* species (Supplementary Table 2). Among these genes, *ndhB* gene possessed the most of RNA editing sites, followed by *ndhD* and *rpoB* genes. The *ndhD, ndhA, ndhF, rpoC1*, and *rpoC2* genes revealed a fraction of variation among 24 *Nicotiana* species (Figure 1C). The serine (S) to leucine (L) conversions were the most frequent, followed by Proline (P) to leucine (L) and serine (S) to phenylalanine (F) conversions (Figure 1D). These changes helped in the formation of hydrophobic amino acids.

### Variant Sites and Highly Variable Regions Analysis

**Figure 2:**
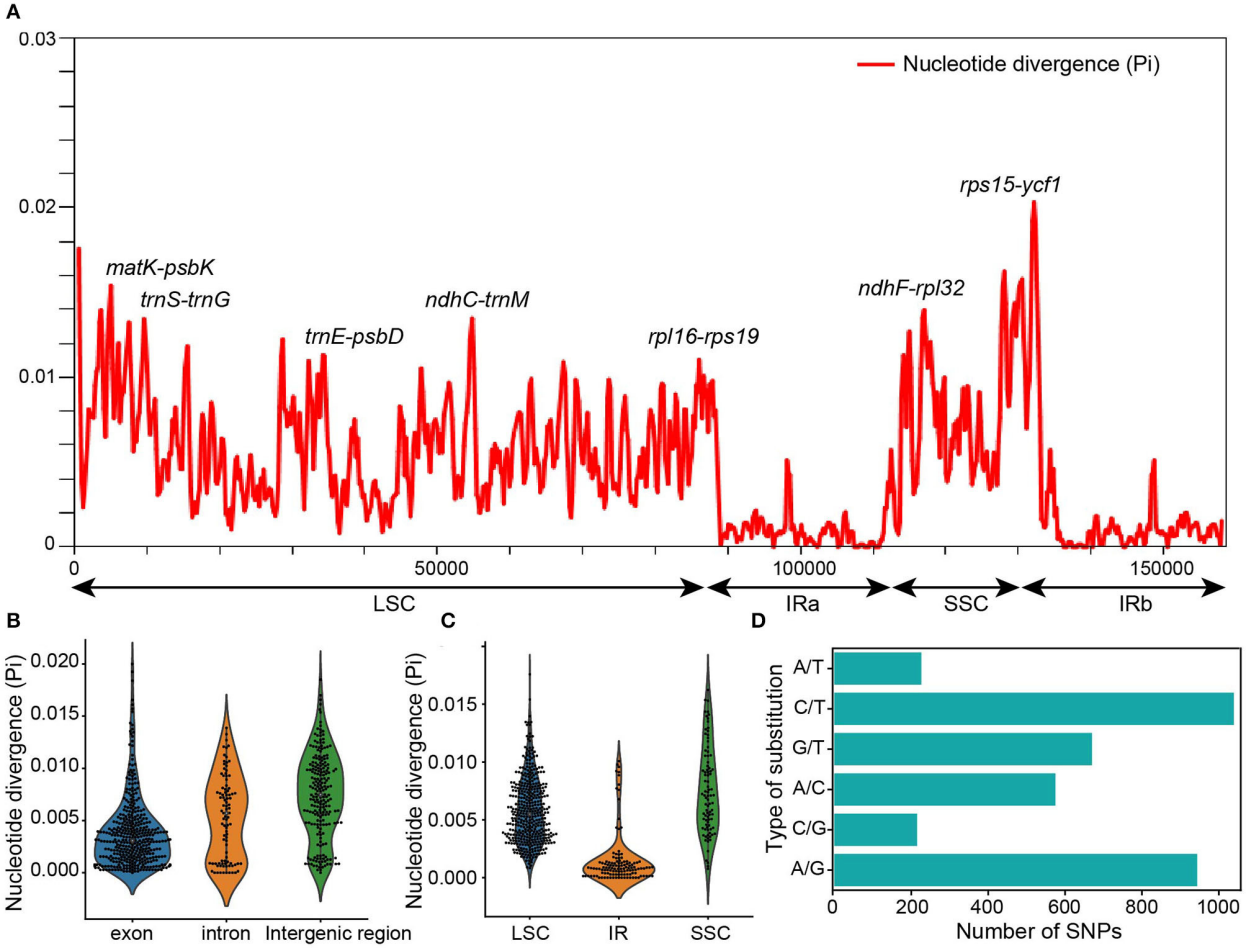
Nucleotide divergence analysis among the chloroplast genomes of *Nicotiana* species. **(A)** Nucleotide diversity by sliding window analysis in the aligned whole chloroplast genomes. Window length: 600 bp, step size: 200 bp. **(B)** The nucleotide diversity values in the LSC, SSC, and IR regions of the chloroplast genomes among *Nicotiana* species. **(C)** The nucleotide diversity values in the CDS, intron, and intergenic regions of the chloroplast genomes among *Nicotiana* species. **(D)** Comparison of substitution in chloroplast genomes of *Nicotiana* species.

The variant sites were determined in *Nicotiana* species using the chloroplast genome of *N. sylvestris* as reference. Among the 24 *Nicotiana* chloroplast genomes, a total of 4,382 variant sites, including 3,882 single nucleotide polymorphisms (SNPs) and 500 small insertions and deletions (InDels), had been identified. In the identified SNPs, the frequencies of transitions (25.5% in A/G and 28.1% in C/T, respectively) were higher than those of transversions (15.5% in A/C, 6.1% in A/T, 5.8% in C/G and 18.1 G/T, respectively) (Figure 2D). As for the transition, similar amounts of A/G (944) and C/T (1,039) were found. While similar amounts of A/T (226) and C/G (215) and amounts of G/T (670) and A/C (575) were also found. Most of the SNPs were distributed within the LSC region (2,781, representing 3.21% of the LSC sequences), but the SSC region contained the highest proportion of SNPs (851, 4.58%), while the variant sites content of the IRa/b regions was the lowest (125 each, 0.49%) (Supplementary Table 3; Figure 1A). In addition, the distribution of InDels among the chloroplast genomes was very similar to that of SNPs: most of the InDels were found in the LSC region (395) followed by the SSC region (53) followed by the IR region (26 each) (Supplementary Table 3).

Additionally, we examined the number of nucleotide diversity (Pi) across the chloroplast genomes in four regions using DnaSP v6.12.03 (Rozas et al., 2017). The results showed that the IRs regions were less divergent than the LSC and SSC regions. The number of nucleotide diversity sites was 4,217 across the chloroplast genomes, 2,863 in the LSC region, 802 in the SSC region, and 211 in the IR regions (Supplementary Table 4). The IR regions showed the lowest nucleotide diversity (Pi = 0.00149), while the SSC region had the highest nucleotide diversity (Pi = 0.00765). The result of the nucleotide diversity analysis was consistent with the results of SNPs and InDels: almost the entire IR regions were conserved, while the LSC and SSC regions were more variable (Figure 2A; Supplementary Table 3).

To detect highly variable regions among the exon, intron, and intergenic regions of chloroplast genomes, we also conducted a sliding window analysis using DnaSP. The sliding window analysis showed that the intergenic regions had greater divergence than the exon and intron regions, and the intron regions had greater divergence than the exon regions (Figure 2B). The number of variable sites was 1,801 in the exon region, 625 in the intron region, and 1,884 in the intergenic regions (Supplementary Table 4). The exon region showed the lowest nucleotide diversity (Pi = 0.00381), while the intergenic region had the highest (Pi = 0.00747).

The sliding window analysis of whole chloroplast genomes revealed seven highly variable regions with Pi ranging from 0.01007 to 0.02031 across 24 complete chloroplast genomes (Figure 2A). The highly variable regions comprised the intergenic regions: *matK-psbK, rps15-ycf1, ndhF-rpl32, trnS-trnG, ndhC-trnM, trnE-psbD, rpl16-rps19*. Among the seven highly variable regions, five regions were in the LSC, and two regions (*rps15-ycf1, ndhF-rpl32*) were in the SSC. None of the hypervariable regions were in the IRs, which had more conserved sequences (Figure 2C).

### Repetitive Sequences Analysis

**Figure 3:**
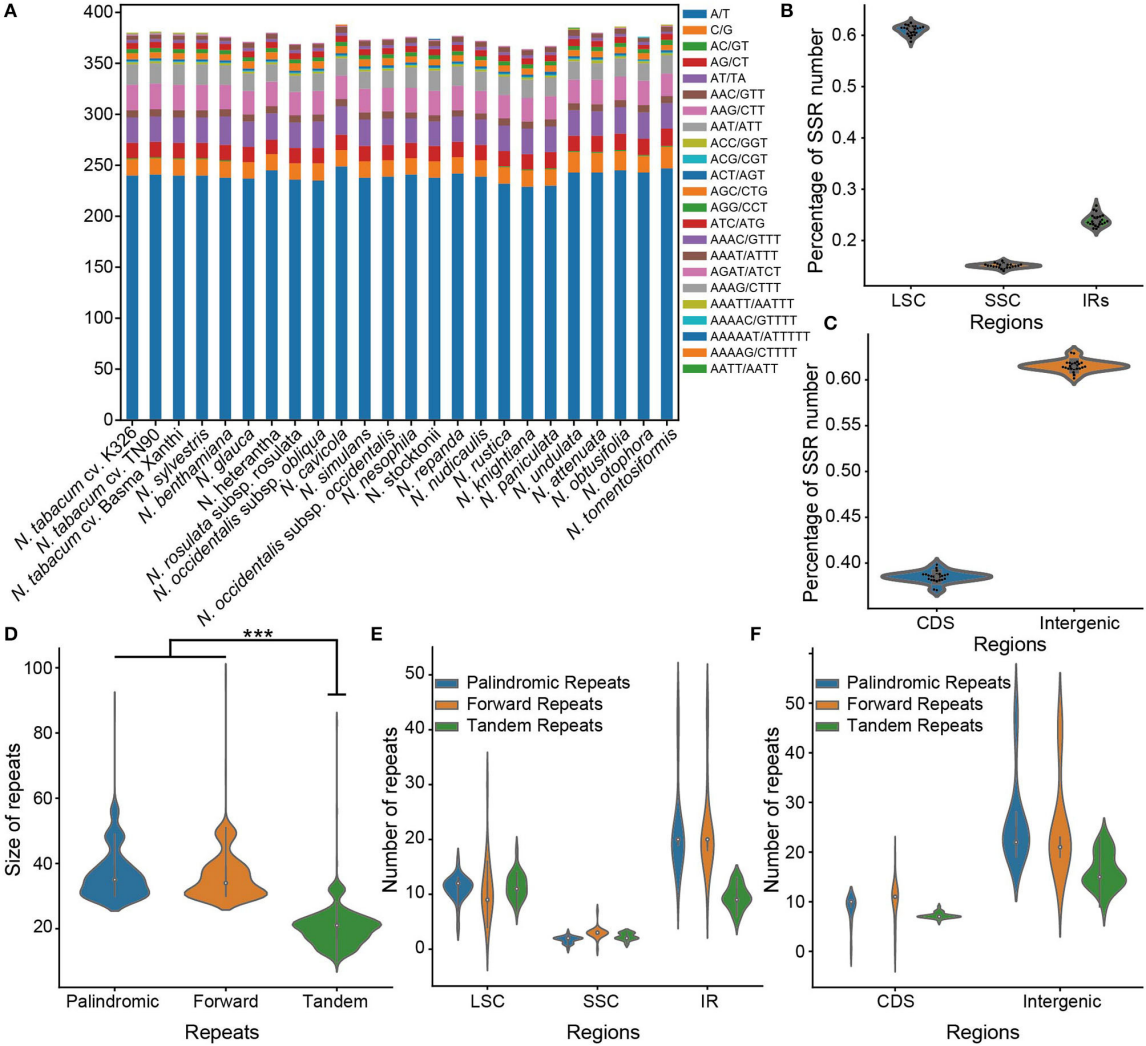
Comparison of sample sequence repeats (SSR) and repeats among 24 chloroplast genomes of *Nicotiana*. **(A)** The number of SSRs among *Nicotiana* species. Each color represented the number of SSR belonging to a specific type, as provided. **(B)** The percentage of SSR number located in different chloroplast regions (LSC: large single-copy, SSC: Small single-copy, IR: inverted repeat region including IRa and IRb) of *Nicotiana* species. **(C)** The percentage of SSR number on CDS and intergenic regions of *Nicotiana* chloroplast genomes. **(D)** The size distribution of palindromic, forward and tandem repeats among the chloroplast genomes. **(E)** The number of repeats among the different regions (LSC, SSC, and IRs) of chloroplast genomes. **(F)** The number of repeats among the CDS and intergenic regions of chloroplast genomes.

We detected and analyzed the occurrence, type, and distributions of chloroplast microsatellites (SSRs) in the 24 *Nicotiana* chloroplast genomes using MISA (Beier et al., 2017). The numbers of SSRs ranged from 364 SSRs in *N. knightiana* to 388 SSRs in *N. tomentosiformis* (Figure 3A; Supplementary Table 5). Among these SSR repeats, mononucleotide repeats (67.03–69.07%) and tetranucleotide repeats (17.53–19.21%) were the most common, followed by dinucleotide repeats (10.37–11.70%) (Supplementary Table 6). The proportions of tri-, penta-, and hexanucleotide repeats were relatively low for each sample. Among mononucleotide repeats, poly A/T (63.65%) repeats were the most common, while poly C/G (4.43%) repeats were less frequent (Supplementary Table 5; Figure 3A). Of all SSRs, 15 were shared across all 24 chloroplast genomes of the genus *Nicotiana* in this study.

In addition, the SSRs were non-randomly distributed in the chloroplast genomes of the genus *Nicotiana*. Of all SSRs, 58.76–62.43% were located within the LSC region, while only 14.09–16.05% and 22.28–26.80% were located within the SSC and IR regions, respectively (Figure 3B; Supplementary Table 7). Similarly, most SSRs also occurred within the intergenic regions (60.16–62.95%) compared to the CDS regions (37.05–39.84%) (Figure 3C; Supplementary Table 7).

For repeat sequences analysis, 13–25 palindromic repeats, 13–29 forward repeats, and 16–30 tandem repeats were identified in the 24 chloroplast genomes of *Nicotiana* species (Supplementary Table 8). Among these, the palindromic repeats had a size of 30–88 bp in length, the forward repeats had a size of 30–97 bp in length, and the tandem repeats had a size of 10–83 bp in length (Figure 3D). Whereas around 77.4% of the palindromic repeats and 80.8% of the forward repeats were 30–40 bp in length, and 76.2% of the tandem repeats were 15–25 bp in length (Figure 3D). In the chloroplast genome regions, the LSC and IR regions held most of the identified repeats, as compared to the SSC regions (Figure 3E). Meanwhile, the repeats existed mostly in the intergenic regions compared with the CDS regions (Figure 3F).

### Phylogenetic Relationship and Divergent Time Estimate

**Figure 4:**
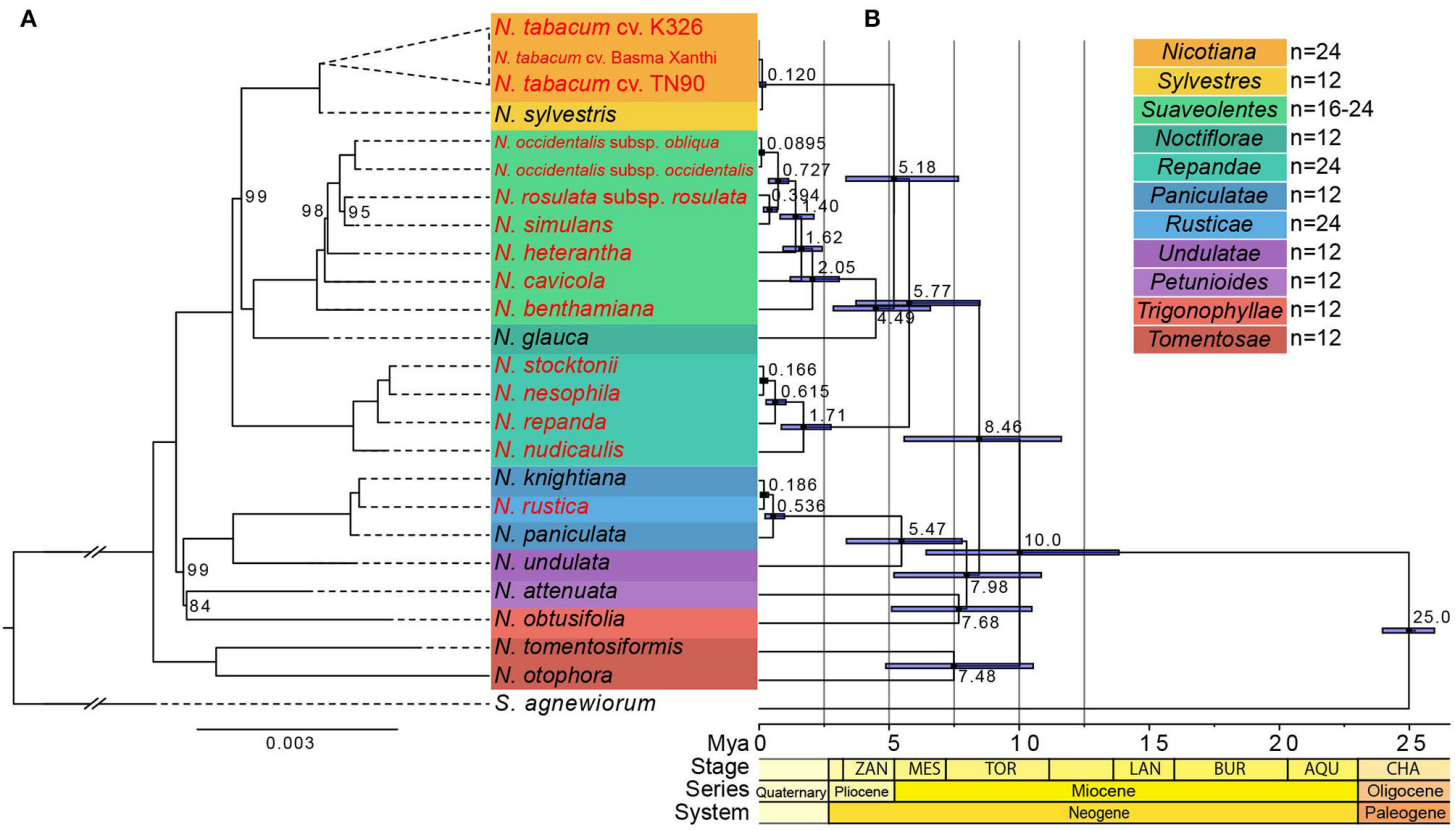
Phylogenetic relationship and divergent time estimate. **(A)** Phylogeny of *Nicotiana* species inferred from maximum likelihood analysis of combined 78 protein-coding sequences. Bootstrap support values <100% were shown above branches. Sections of the genus *Nicotiana* are shown on the right-hand side, corresponding to the shaded boxes. The allotetraploid species were indicated by red fonts. Scale indicated the base substitution per site. The *Nicotiana* sections according to Knapp et al. (2004). **(B)** Divergent time estimate of the 24 *Nicotiana* taxa and one outgroup species based on 78 protein-coding genes. The branch length of the cladogram reflected the divergent time, and the number beside the node denoted the node age, with the purple bar as 95% highest probability density (HPD).

To study the phylogenetic position of the 24 *Nicotiana* species, we used 78 protein-coding sequences shared by the chloroplast genomes for multiple alignments. One species, *Solanum agnewiorum*, was set as outgroups. The maximum likelihood (ML) phylogenetic result was largely in agreement with a previous study (Clarkson et al., 2004) with strong support, and the only substantive point at which they differed corresponded to the placement of sections (Figure 4A). The *Nicotiana* sections were labeled according to Knapp et al. (2004). In this phylogenetic tree, the sections *Tomentosae, Repandae*, and *Suaveolentes* all formed monophyletic groups. Members of section *Tomentosae* (*N. otophora* and *N. tomentosiformis*) was sister to the rest of the genus, which had been observed in the previous study (Clarkson et al., 2004). The next strongly supported clade was composed of sections *Paniculatae, Undulatae, Petunioides, Rusticae*, and *Trigonophyllae*, which was sister to the rest of the genus, excluding section *Tomentosae*. In the clade of section *Paniculatae*, the allotetraploid species *N. rustica* was closer genetically to *N. knightiana* than *N. paniculata*, which differed from the previous studies (Clarkson et al., 2004; Knapp et al., 2004). The last strongly supported clade was composed of sections *Sylvestres, Nicotiana, Suaveolentes, Noctiflorae*, and *Repandae*. In this clade, the allotetraploid species of section *Repandae* including *N. stocktonii, N. nesophila, N. repanda*, and *N. nudicaulis* formed a well-supported monophyletic clade that was successively sister to the rest. Meanwhile, the section *Suaveolentes* composed of *N. benthamiana, N. cavicola, N. heterantha, N. simulans, N. rosulate*, and *N. occidentalis* formed another well-supported monophyletic clade. The allotetraploid species of sections *Suaveolentes* and *Nicotiana* were successively sister to the diploid species of sections *Noctiflorae* and *Sylvestres*, respectively.

For the investigation of divergence times of *Nicotiana* sections and the evolutionary history of polyploids, BEAST analysis based on concatenated datasets of all protein-coding genes in the chloroplast genomes was used for molecular dating (Figure 4B). The *Nicotiana* chloroplast was found to have diverged from the outgroup *S. agnewiorum* at ~25 million years ago (Mya), (95% highest posterior density [HPD]: 23.97–25.96 Mya); The most recent common ancestor (MRCA) of all *Nicotiana* chloroplast haplotypes was estimated at ~10.0 Mya (95% HPD: 6.42–13.83 Mya). Within the genus *Nicotiana*, there was evidence of recent hybrid origins of several polyploid lineages. The tetraploid *N. tabacum* was diverged from the diploid *N. sylvestris* chloroplast haplotype by ~0.12 Mya (95% HPD: 0.02–0.25 Mya). The tetraploid section *Suaveolentes* was diverged from the diploid *N. glauca* by ~4.49 Mya (95% HPD: 2.85–6.58 Mya). The tetraploid section *Repandae* was diverged from the MRCA of diploid *N. glauca* and *N. sylvestris* by ~5.77 Mya (95% HPD: 3.73–8.48 Mya). In addition, the tetraploid *N. rustica* was diverged from the diploid *N. knightiana* by ~0.186 Mya (95% HPD: 0.05–0.36 Mya). The results of molecular dating in the genus *Nicotiana* thus indicated that allopolyploid species (*N. tabacum*, section *Suaveolentes*, section *Repandae, N. rustica*) were formed among ~0.12 million (*N. tabacum* L.) to ~5.77 million (section *Repandae*) years ago (Figure 4B).

## Discussion

### The Molecular Evolution of *Nicotiana* Chloroplast Genomes

The chloroplast genomes have made significant contributions to taxonomic studies of several plant families and resolving evolutionary relationships within phylogenetic clades (Moore et al., 2010; Barrett et al., 2016). This study successfully acquired the 19 complete chloroplast genomes of *Nicotiana* species and performed a comparative analysis among the chloroplast genomes of 24 *Nicotiana* species, subspecies, and varieties of 11 out of the 13 *Nicotiana* sections. The same as those of other land plants (Wicke et al., 2011), the assembled chloroplast genomes of *Nicotiana* species were all the typical quadripartite circular structure consisting of a small single-copy region, a large single-copy region, and a pair of inverted repeats regions. Moreover, the genome organization and size, gene composition and order, as well as GC content also showed high similarity among the *Nicotiana* chloroplast genomes (Table 1), which could be attributed to chloroplast genomes of land plants having a conserved structure (Wicke et al., 2011). The higher GC content observed in the IR regions (Figure 1A) could be a result of the presence of abundant rRNA and tRNA genes, which contained a relatively higher GC content (Figure 1B) (Niu et al., 2017; Menezes et al., 2018; Mehmood et al., 2020).

RNA editing is an important process of gene regulation through nucleotide modification at the post-transcriptional level, which maintains the functional amino acid sequence of the evolutionarily conserved protein (Rodrigues et al., 2017). In higher plant chloroplasts, cytidine to uridine conversion (C-to-U), as the major type of RNA editing, occurs at around 30 specific sites in mRNAs (Sasaki et al., 2003). In this study, the number of predicted RNA editing sites in Nicotiana chloroplast genomes varied from 35 to 38, of which *ndhB* gene possessed most of the RNA editing sites, followed by *ndhD* and *rpoB* genes (Figure 1C). Although it has been reported that several nuclear-encoded proteins containing pentatricopeptide repeat (PPR) motifs have been essential for chloroplast RNA editing, the molecular mechanisms determining the specificity of the RNA editing process are not fully understood (Manna, 2015). More work is needed in this area in the future.

### Genetic Variation and Repeats of *Nicotiana* Chloroplast Genomes

Genome variation and nucleotide diversity in the chloroplast genomes provide useful information for identifying molecular markers, reconstructing phylogenetic relationships, and exploring population genetics in angiosperms (Li C. et al., 2020; Wang et al., 2021). Totally, 3,882 SNPs, 500 InDels, and 4,217 nucleotide variability sites have been identified in the 24 chloroplast genomes of *Nicotiana* species. Among SNPs, A/G and C/T conversions were most abundant as compared to other SNPs (Figure 2D). The comparative analysis of variation information in different regions indicated that the IR regions were highly conserved compared to the LSC and SSC regions, as reflected by the fact that <6.5% of the SNPs that had been identified in this study were located within the IR regions even though IR regions constituted about one-third of the chloroplast genomes (Figures 1A, 2A; Supplementary Table 3). The low level of variant sites and nucleotide diversity observed in the IR regions was very common among plant chloroplast genomes (Choi et al., 2016; Wang et al., 2019, 2022; Sun et al., 2022). In addition, the intergenic regions had greater divergence than the exon and intron regions (Figure 2C; Supplementary Table 8). Similar results have been shown in other chloroplast genomes of angiosperms (Liu et al., 2019; Li L. et al., 2020). The variation hotspot regions of chloroplast genomes could be used to develop accurate and cost-effective molecular markers for population genetics, DNA barcoding, and evolution studies (Dong et al., 2012; Song et al., 2017; Amar, 2020). Previously, the markers of *trnL-F* spacer, *trnS-G* spacer, *ndhF*, and *matK* had been used for the molecular phylogeny of *Nicotiana* species (Aoki and Ito, 2000; Clarkson et al., 2004). Here, seven hotspot regions (*matK-psbK, rps15-ycf1, ndhF-rpl32, trnS-trnG, ndhC-trnM, trnE-psbD, rpl16-rps19*) of chloroplast genomes were discovered by sliding window analysis (Figure 2A). The results indicated that these hypervariable regions may have better resolution for species identification than the nucleotide sequences previously used (Clarkson et al., 2004).

Repeats, including SSRs, palindromic repeats, forward repeats, and tandem repeats, in the chloroplast genomes provide useful information for evolutionary studies and play a vital role in recombination and rearrangement of the genome, genetic diversity, and biogeography within and between groups (Bi and Liu, 1996; Hokanson et al., 1998; Triest, 2008). In this study, a total of 364–388 SSRs, 13–25 palindromic repeats, 13–29 forward repeats, and 16–30 tandem repeats were detected throughout the 24 chloroplast genomes of *Nicotiana* species, among which the mononucleotide repeats were the most common representing 68.08% of the total number of SSRs (Figure 3A; Supplementary Table 5). The LSC region contained a higher amount of SSRs and tandem repeats in comparison to the SSC and IR regions, while the SSC region had the highest density of SSRs and the least amount of palindromic repeats and forward repeats (Figure 3B; Supplementary Table 8). In addition, the intergenic regions also had a more considerable amount of SSRs, palindromic repeats, forward repeats and tandem repeats compared with the CDS regions (Figure 3C). Similar to the results has also been demonstrated in other studies of angiosperm chloroplast genomes (Li and Zheng, 2018; Asaf et al., 2021). Nevertheless, still substantial genetic variation, SSR loci, and nucleotide variability across the chloroplast genomes have been identified among the 24 chloroplast genomes of the genus *Nicotiana*, which may serve as useful data for future studies.

### Phylogenetics and the Origins of Allotetraploid in Genus *Nicotiana*

Polyploidy is common in the genus *Nicotiana*, with ~40% of species being allotetraploid (Knapp et al., 2004). Although parental lineages of most allotetraploid *Nicotiana* species have been widely established (Aoki and Ito, 2000; Clarkson et al., 2004, 2005, 2010; Leitch et al., 2008; Lee et al., 2016; Mehmood et al., 2020), few studies have distinctly demonstrated the polyploidization events, except the allotetraploid *N. tabacum* (2*n* = 4*x* = 48). The section *Nicotiana* was postulated to have evolved as an amphiploid derivative ~0.2 Mya through an interspecific hybrid between the ancestor species of *N. sylvestris* (maternal donors, 2*n* = 2*x* = 24) and *N. tomentosiformis* (paternal donors, 2*n* = 2*x* = 24) (Yukawa et al., 2006; Sierro et al., 2013). Based on the chloroplast phylogeny, the position of allopolyploid species might reflect its maternal lineages, as the chloroplast is maternally inherited in the genus *Nicotiana* (Avni and Edelman, 1991).

Here, the phylogenetic backbone structure constructed with 78 protein-coding sequences of chloroplast genomes was substantially consistent with the structure based on molecular markers in the previous studies (Clarkson et al., 2004; Leitch et al., 2008), except for a slight conflict in several sections (Figure 4A). The phylogenetic tree showed that the allotetraploid section *Nicotiana* was successively sister to the diploids *N. sylvestris*, which was identical to the previous result (Yukawa et al., 2006). However, the parental genome donors for the allopolyploid *N. rustica* (2*n* = 4*x* = 48) were still controversial and unresolved. Several previous studies based on the chloroplast DNA regions (Aoki and Ito, 2000; Clarkson et al., 2004), ITS loci (Komarnyts'kyi et al., 1998), random amplified polymorphic DNA (RAPD) (Khan and Narayan, 2007), and genomic *in situ* hybridization (GISH) (Lim et al., 2005) showed that the *N. rustica* was a natural allotetraploid through interspecific hybridization between the ancestor species of *N. paniculata* (or the common ancestor of the sister pair, *N. knightiana/N. paniculata*) and *N. undulata*. Our chloroplast phylogenetic result showed that *N. knightiana* is more closely related to *N. rustica* than *N. paniculata* (Figure 4A), which indicated the progenitors of extant *N. knightiana* might have donated the maternal genome of *N. rustica*. Similar observations were reported in the recent studies (Sierro et al., 2018; Mehmood et al., 2020). Section *Repandae* (2*n* = 4*x* = 48) consists of four allopolyploid species: *N. nudicaulis, N. repanda, N. stocktonii*, and *N. nesophila* formed monophyletic group, which indicated that all these species descended from a single common ancestral allopolyploid species (Figure 4A). Previous phylogenetic relationships inferred from multiple chloroplast DNA regions (Clarkson et al., 2004) and nuclear ribosomal DNA (rDNA) (Clarkson et al., 2005) indicated that an ancestor of *N. sylvestris* was the maternal genome donor of section *Repandae*. Whereas, our phylogenetic result shows that the common ancestor of *N. sylvestris* and *N. glauca* might have donated the maternal genome of section *Repandae* (Figure 4A). In section *Suaveolentes*, as the largest group of allotetraploid of native Australian *Nicotiana* species (Knapp et al., 2004), polyploid evolution has been accompanied by changes in chromosome number, probably through diploid reductions *via* chromosome deletions or fusions (2n ranges from 32 to 48) (Leitch et al., 2008). However, the parental lineages of section *Suaveolentes* have been problematic to identify (Kelly et al., 2013). Recent research had shown that a member of the section *Sylvestres* lineage acted as the paternal progenitor and a member of either section *Petunioides* or section *Noctiflorae* that also contained introgressed DNA from the other, or a hypothetical hybrid species between these two sections, was the maternal progenitor (Kelly et al., 2013) whereas our phylogenetic tree supported that the diploid section *Noctiflorae* lineages (*N. glauca*) acted as the most likely maternal progenitor of section *Suaveolentes* (Figure 4A), which was consistent with a previous study (Clarkson et al., 2004).

Owing to the different database and phylogenetic structures, the divergence time of polyploids was not completely consistent with the previous studies (Clarkson et al., 2005; Leitch et al., 2008; Mehmood et al., 2020; Schiavinato et al., 2020), which suggested the hybridization events at an age of <0.2 Mya for section *Nicotiana* and *N. rustica*, ~4.5 Mya for section *Repandae*, and more than 10 Mya for section *Suaveolentes* as the oldest polyploids, respectively. Our result shows that the polyploid species range considerably from ~0.12 million (section *Nicotiana*) to ~5.77 million (section *Repandae*) years ago (Figure 4B). The younger polyploids (*N. tabacum* and *N. rustica*) were estimated to have arisen at ~0.120 Mya and ~0.186 Mya, respectively. The older polyploids (section *Repandae* and *Suaveolentes*) were considered to have originated from a single polyploid event at ~5.77 Mya and ~4.49 Mya, respectively, followed by speciation to produce an abundance of polyploid species known today.

## Conclusion

In this study, we analyzed and compared the structural characteristics of 24 chloroplast genomes of *Nicotiana* species, and inferred the phylogenetic divergence time. The chloroplast genomes of *Nicotiana* have a typical quadripartite structure, including 78 protein-coding genes, 20 tRNA genes, and four rRNA genes, with a total length of 155,327-156,142 bp. We found seven mutation hotspots, which could be used as potential DNA barcodes in the future phylogenetic study of *Nicotiana*. Phylogenetic relationships based on combined protein-coding genes showed that the progenitors of diploid *N. sylvestris, N. knightiana*, and the common ancestor of *N. sylvestris* and *N. glauca* might have donated the maternal genomes of allopolyploid *N. tabacum, N. rustica*, and section *Repandae*, respectively. Meanwhile, the diploid section *Noctiflorae* lineages (*N. glauca*) acted as the most likely maternal progenitor of section *Suaveolentes*. Reconstructing the divergence time of *Nicotiana* shows that the polyploid events range considerably from ~0.12 million (section *Nicotiana*) to ~5.77 million (section *Repandae*) years ago. The younger polyploids (*N. tabacum* and *N. rustica*) were estimated to have arisen at ~0.120 Mya and ~0.186 Mya, respectively. The older polyploids (section *Repandae* and *Suaveolentes*) were considered to have originated from a single polyploid event at ~5.77 Mya and ~4.49 Mya, respectively. These chloroplast genomes contribute to the study of genetic diversity and species evolution of *Nicotiana* while providing useful information for taxonomic and phylogenetic studies of *Nicotiana*. In the future, we will expand genomic sampling, including nuclear genomes, to comprehensively compare and discuss the phylogeny and polyploid speciation of the *Nicotiana* species.

## Data Availability Statement

The datasets presented in this study can be found in online repositories. The names of the repository/repositories and accession number(s) can be found in the article/Supplementary Material.

## Author Contributions

SW and JG conceived and designed the study. SW and HC conducted the bioinformatics analysis. ZL, WP, and YW assisted in data collection. SW, HC, and MC wrote and revised the manuscript. All authors read and approved the final manuscript.

## Funding

This work was supported by the National Natural Sciences Foundation of China [32070677]; the 151 Talent Project of Zhejiang Province (first level); Jiangsu Collaborative Innovation Center for Modern Crop Production and Collaborative Innovation Center for Modern Crop Production cosponsored by the province and ministry; the key funding of CNTC [110202101003 (JY-03)]. This study received funding from NNSFC (No. 32070677) and CNTC (No. 110202101003 (JY-03)). The funder was not involved in the study design, collection, analysis, interpretation of data, the writing of this article or the decision to submit it for publication.

## Conflict of Interest

SW, JG, WP, and YW were employed by China Tobacco Hunan Industrial Corporation. The remaining authors declare that the research was conducted in the absence of any commercial or financial relationships that could be construed as a potential conflict of interest.

## Publisher's Note

All claims expressed in this article are solely those of the authors and do not necessarily represent those of their affiliated organizations, or those of the publisher, the editors and the reviewers. Any product that may be evaluated in this article, or claim that may be made by its manufacturer, is not guaranteed or endorsed by the publisher.
